# More passive internal tibial rotation with posterior cruciate ligament retention than with excision in a medial pivot TKA implanted with unrestricted caliper verified kinematic alignment

**DOI:** 10.1007/s00167-021-06840-0

**Published:** 2021-12-18

**Authors:** Alexander J. Nedopil, Stephen M. Howell, Maury L. Hull

**Affiliations:** 1 Department of Orthopaedic Surgery, König-Ludwig-Haus, University of Würzburg, Würzburg, Germany; 2 Department of Biomedical Engineering, University of California at Davis, 451 E. Health Sciences Drive, Room 2303, Davis, CA 95616 USA

**Keywords:** Total knee replacement, Total knee arthroplasty, Calipered, Posterior cruciate ligament, Tibial rotation, Insert thickness

## Abstract

**Purpose:**

Excision of the posterior cruciate ligament (PCL) is recommended when implanting a medial pivot (MP) total knee arthroplasty (TKA) to reduce the risk of limiting flexion by over-tensioning the flexion space. The present study determined whether PCL retention (1) limits internal tibial rotation and (2) causes anterior lift-off of the insert in 90° flexion after implantation of an MP design with unrestricted caliper verified kinematic alignment (KA).

**Methods:**

Four surgeons implanted an MP TKA design with medial ball-in-socket and lateral flat tibial insert in ten fresh-frozen cadaveric knees. Before and after PCL excision, trial inserts with medial goniometric markings measured the angular I–E tibial orientation relative to the trial femoral component's medial condyle in extension and at 90° flexion, and the surgeon recorded the occurrence of anterior lift-off of the insert at 90° flexion.

**Results:**

PCL retention resulted in greater internal tibial rotation than PCL excision, with mean values of 15° vs 7° degrees from maximum extension to 90° flexion, respectively (*p* < 0.0007). At 90° flexion, no TKAs with PCL retention and one TKA with PCL excision had anterior lift-off of the insert (N.S.).

**Conclusions:**

This preliminary study of ten cadaveric knees showed that PCL retention restored more passive internal tibial rotation than PCL excision with a negligible risk of anterior lift-off. However, in vivo analysis from multiple authors with a larger sample size is required to recommend PCL retention with an MP TKA design implanted with unrestricted caliper verified KA.

## Introduction

An ongoing debate in total knee arthroplasty (TKA) is whether to retain or excise the posterior cruciate ligament (PCL). Because PCL excision predictably increases laxity in the flexion space an unpredictable amount, two categories of implant design evolved to compensate for flexion instability: the medial pivot (MP) and the posterior stabilized (PS) cam-and-post mechanism [8, 11, 16, 36]. In addition, PCL excision is often used with mechanically aligned (MA) TKA to treat an over-tensioned PCL and flexion space as most femoral and tibial components deviate from the patient's pre-arthritic joint lines which can cause anterior lift-off of the insert at 90° flexion [12, 16, 32, 36].

Although the increase in flexion space laxity from PCL excision is well studied, the adverse effect on internal rotation of the tibia with flexion after TKA is less clear. In the native (i.e., healthy knee), the inherent PCL tension drives the tibia's internal rotation with knee flexion, which obligatorily decreases the Q-angle optimizing the retinacular ligaments' tension that guides patellofemoral tracking during knee flexion [4, 7, 18]. In TKA, restoring native internal rotation of the tibia could minimize the risks of patellar tilt, lateral displacement, and anterior knee pain, which the patient needs to experience high satisfaction [3, 19].

For the native knee, the reported mean value of internal tibial rotation from maximum extension to 90° flexion is 18° [4, 5, 10]. When performing TKA, this arc of tibial motion is desirable.

**Fig. 1:**
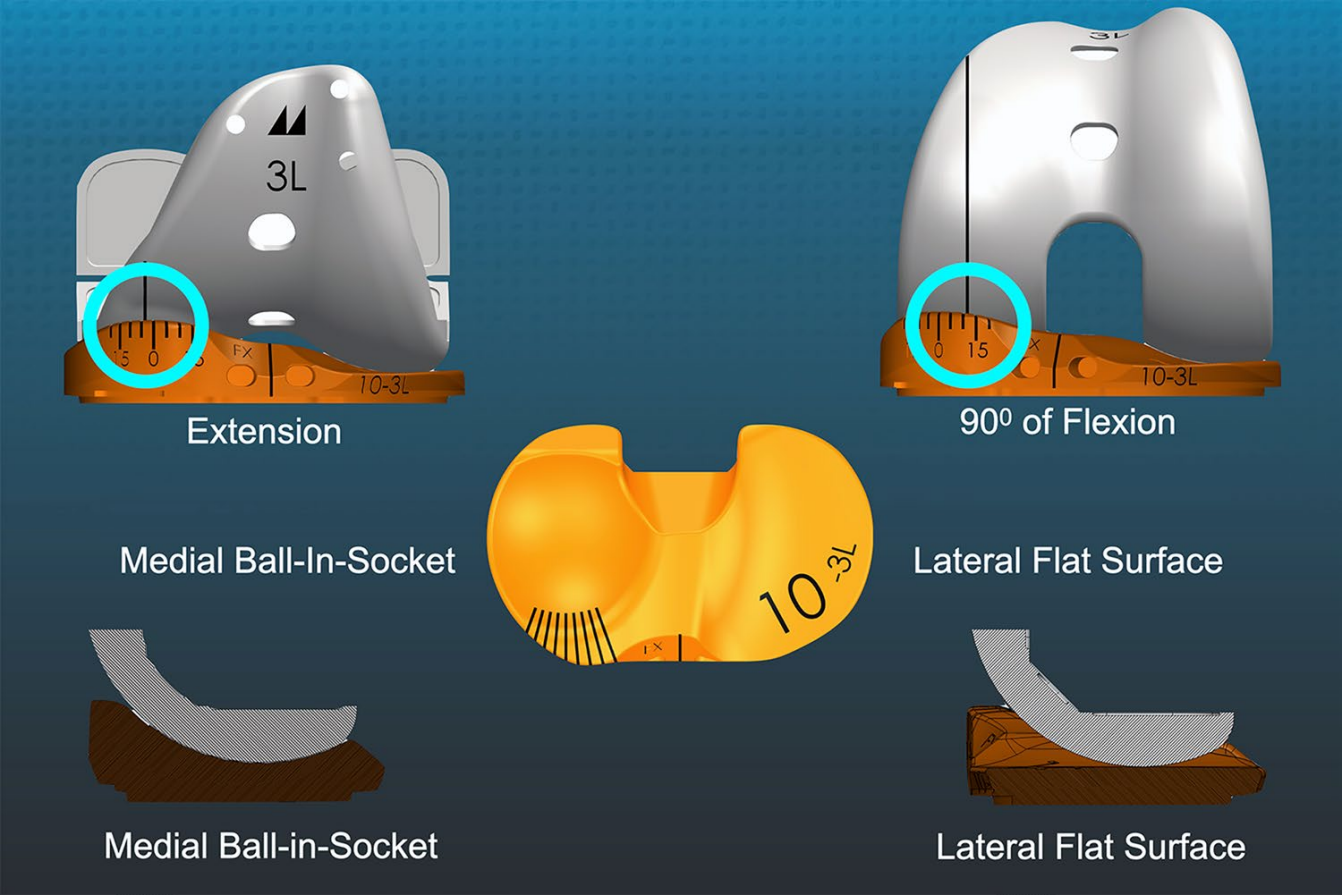
Schematics show a left trial insert goniometer with an anteromedial scale in 5° intervals. The I–E rotation of the tibia relative to a longitudinal line on the medial condyle of the trial femoral component (circles) was 3° external in extension and 12° internal at 90° of flexion resulting in 15° of internal rotation like the native knee. The medial pivot insert is composed of a medial ball-in-socket and a lateral flat articular surface

**Fig. 2:**
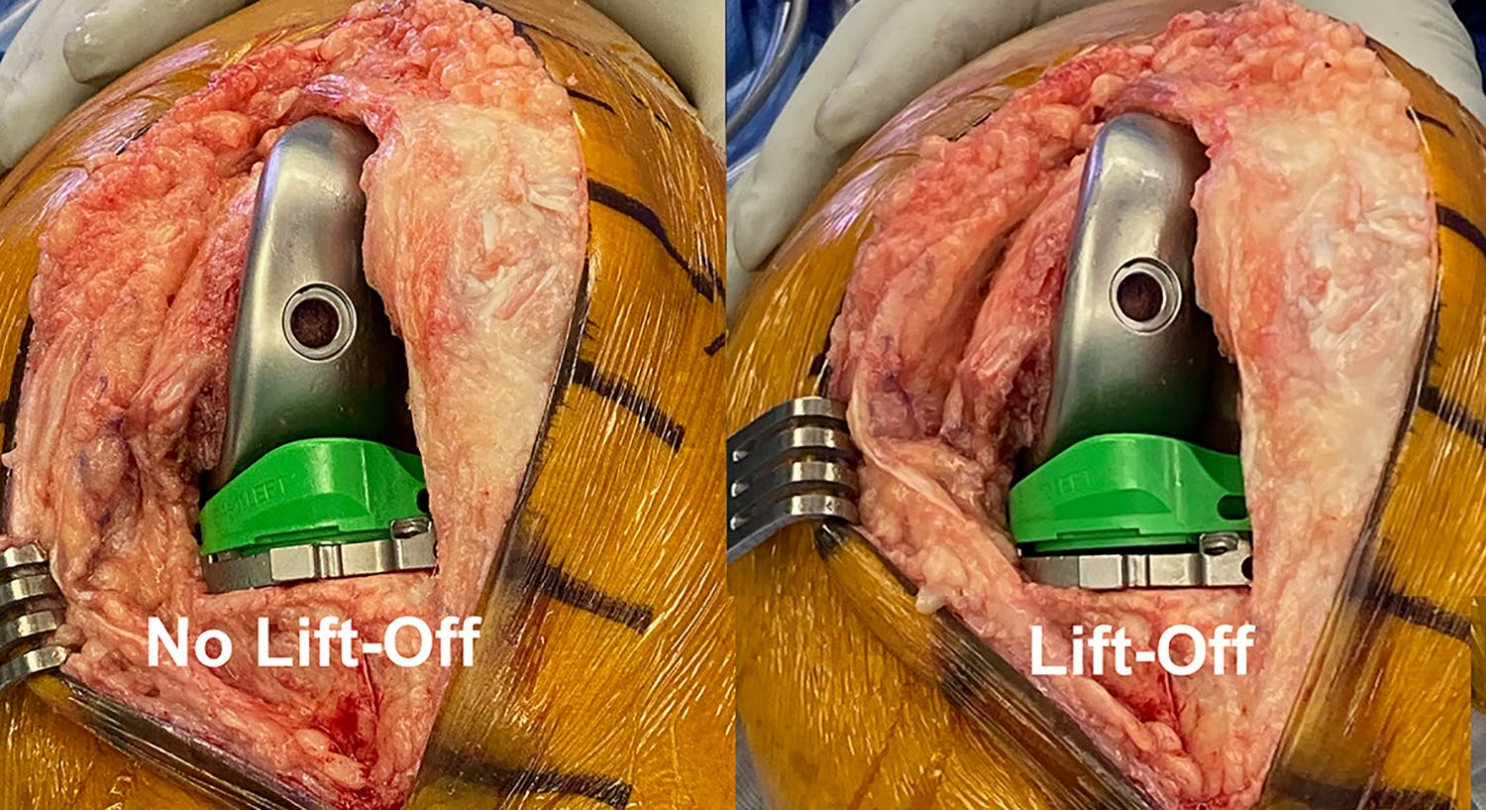
Intraoperative photographs of a left TKA in 90° flexion show examples of no anterior lift-off and lift-off of the trial insert (not goniometric) from the trial baseplate, which indicates an over-tensioned flexion space and PCL

Two signs of an over-tight flexion space are loss of passive internal rotation of the tibia relative to the femur and anterior lift-off of the insert. A trial insert goniometer can determine the tibial orientation relative to a longitudinal line on the trial femoral component's medial condyle. The difference in orientation between flexion angles computes internal–external (I–E) rotation over a motion arc (Fig. 1) [20, 23]. The occurrence of anterior lift-off or 'booking' of the trial insert (or baseplate) indicates an over-tight flexion space (Fig. 2) [32].

Accordingly, this study of ten cadaveric knees evaluated a MP TKA design with a medial ball-in-socket and flat lateral insert implanted with unrestricted caliper verified kinematic alignment (KA), which restores the patient’s pre-arthritic joint lines and native knee posterior laxity and medial and lateral tibial compartment forces [28, 29, 33–35]. The first hypothesis was that PCL retention would restore internal tibial rotation between extension and 90° flexion closer to reported mean values for the native knee than PCL excision. The second hypothesis was that PCL retention would not over-tension the TKA as indicated by a low occurrence of anterior lift-off of the insert at 90° flexion. Proof of these hypotheses would support retaining the PCL when implanting the MP TKA design with unrestricted caliper verified KA.

## Methods and materials

A registered tissue bank provided 12 fresh-frozen lower limb specimens from human donors for the study. An orthopedic surgeon screened anteroposterior and lateral radiographs of the knee and selected ten specimens that had no more than a Grade I or II Kellgren–Lawrence arthritis classification. One knee was studied in two female and eight male Caucasian donors with a mean age of 70 ± 19 years (29–88) and a mean BMI of 28 ± 5 kg/m^2^. Five knees were operated on by one orthopedic surgeon, two knees by another, and three knees by one of three surgeons. According to the University of California at Davis policies, this study did not require institutional review board (IRB) approval since the cadaveric specimens were de-identified. A power analysis determined that a matched pair analysis of nine specimens could detect a minimum difference of a 5° change in internal rotation between TKA’s with and without PCL retention assuming a Type I error (alpha) of 0.05, a standard deviation of 5° (i.e., effect size = 1), and a power (1−beta) of 0.85.

The study evaluated a MP TKA designed by Freeman and Pinskerova with a medial ball-in-socket conformity and a flat lateral insert (Fig. 1) [4, 5, 26, 27]. The insert had a posterior cut-out for retention of the PCLwith surrounding bone island. The tibial baseplate had an anatomic footprint that, when best-fit to the tibial resection, set the anterior–posterior (A–P) orientation parallel to the flexion–extension (F–E) plane of the pre-arthritic knee (GMK Sphere, Medacta International, www.medacta.com) [25]. The patella was left intact.

### Surgical technique

**Fig. 3:**
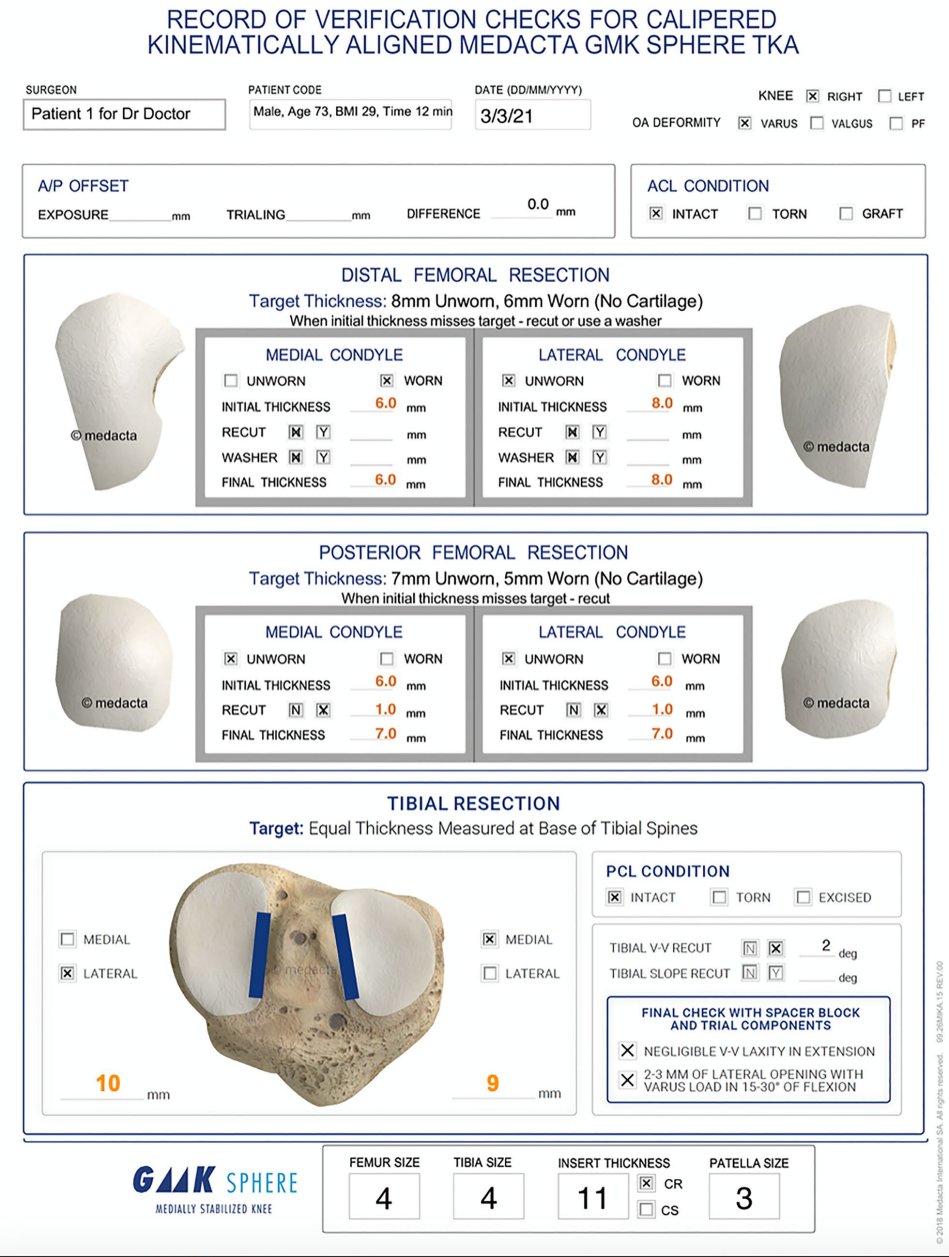
Worksheet for intraoperatively recording serial verification checks for a right knee with a varus deformity showing the caliper measurements of bone resections for a femoral component with a 9 mm-thick distal femoral condyles and 8 mm-thick posterior femoral condyles. The thicknesses of the distal and posterior femoral resections are adjusted so that they equal the thickness of the component within 0 ± 0.5 mm after compensating for 2 mm of cartilage wear when present and a ~ 1 mm kerf from the saw cut

**Fig. 4:**
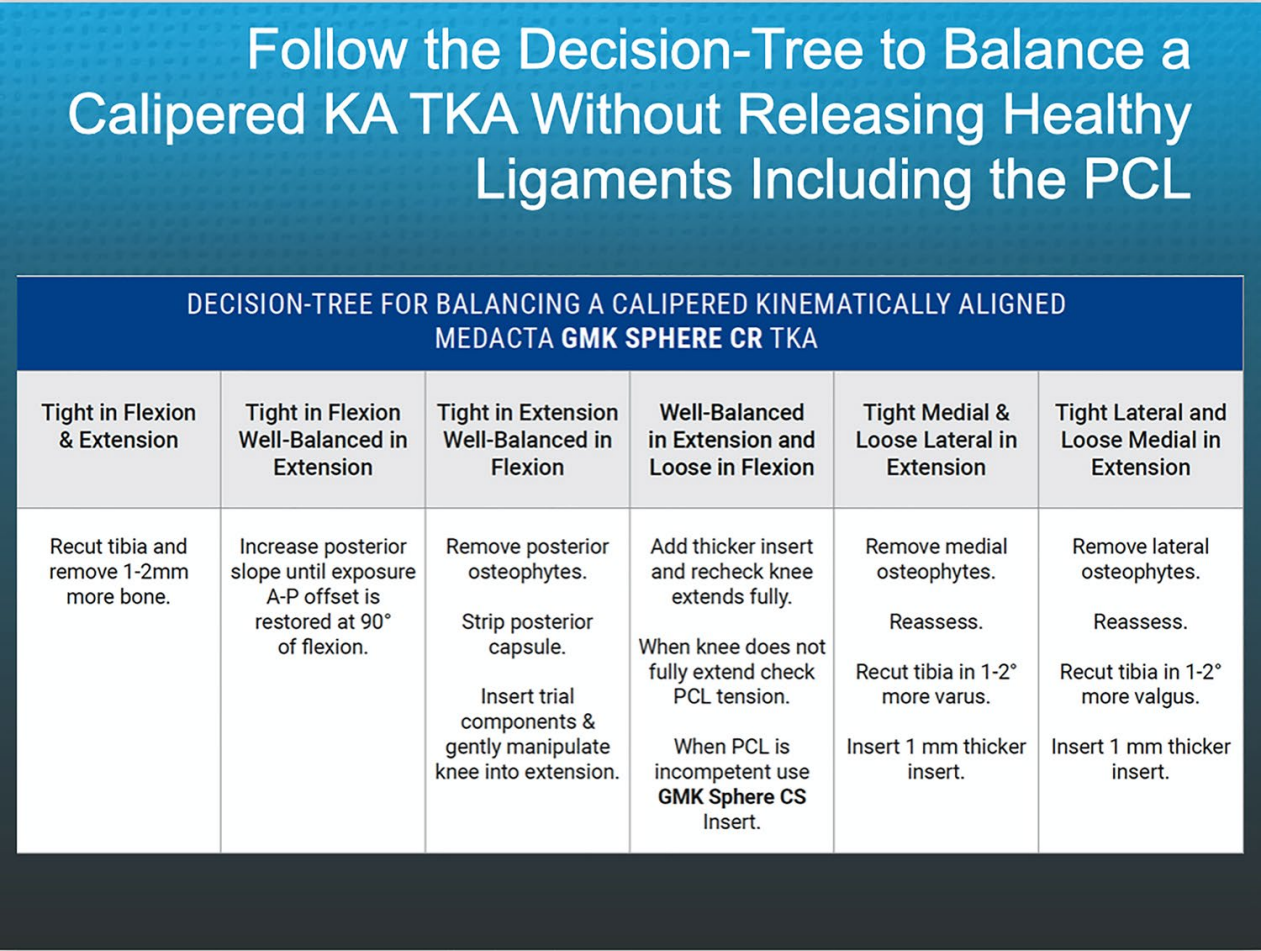
Composite shows the decision-tree followed by the surgeons when they performed unrestricted caliper verified KA TKA. The technique sets the components to restore the pre-arthritic distal and posterior femoral and proximal tibial joint lines within 0 ± 0.5 mm, which restores native tibial compartment forces without the release of healthy ligaments, including the PCL [29, 33, 34]

The following is an overview of the previously described unrestricted caliper verified KA technique performed through a mid-vastus approach using intraoperatively recorded verification checks and following a decision-tree (Figs. 3, 4) [14]. For the femoral component, the varus–valgus (V–V) and I–E orientations and the A–P and proximal–distal (P–D) positions were set coincident with the patient’s pre-arthritic distal and posterior joint lines by adjusting the calipered thicknesses of the distal and posterior femoral resections to within 0 ± 0.5 mm of those of the femoral component condyles after compensating for cartilage wear and the kerf of the saw blade. An accuracy analysis showed these steps restore the distal lateral femoral joint line of 97% of patients within the normal left to right symmetry and set the I–E orientation of the femoral component with a deviation of 0.3° (external) ± 1.1° from the KA target of the F–E plane of the patient’s knee [13, 21, 22, 24].

The surgeon followed six options in a decision-tree to set the V–V and posterior slope orientation and thickness of the tibial component to restore the patient’s pre-arthritic tibial joint line and limb alignment and balance the knee by restoring the native tibial compartment forces (Fig. 4) [20, 29, 33, 35]. The varus–valgus orientation of the tibial resection was adjusted working in 1°–2° increments until there was negligible medial and lateral lift-off from the femoral component during a varus–valgus laxity assessment in extension with the spacer block and trial tibial insert. An accuracy analysis showed that these steps restore the proximal tibial joint line of 97% of patients within native left to right symmetry [13, 15, 24]. The posterior slope was adjusted by setting an angel wing inserted through the medial slot of the tibial guide parallel to the patient’s pre-arthritic slope. An accuracy analysis showed a 0° mean difference between the tibial component's posterior slope and the patient’s pre-arthritic posterior slope [15, 20]. A best-fit of the largest anatomically shaped trial tibial baseplate inside the cortical rim of the tibial resection set the I–E orientation and A–P and medial–lateral (M–L) positions. An accuracy analysis showed a mean 2° (external) ± 5° deviation of the I–E orientation of the tibial component from the KA target of the F–E plane of the patient’s knee [21, 24, 25, 28, 33, 35].

The following steps determined the optimal insert thickness. The knee was placed in 90° of flexion and the PCL was palpated to verify that it was intact. A goniometric tibial insert that matched the thickness of the spacer block was positioned. The knee was placed in maximum extension and the surgeon verified that it hyperextended like the pre-arthritic knee. When the knee had a flexion contracture, a 1 mm thinner insert was used. The surgeon checked the V–V laxity and verified that it was negligible in full extension and had a 3–4 mm lateral gap and negligible medial gap with the knee in 15°–30° of flexion. The knee was placed in 90° of flexion and the surgeon verified that the passive I–E rotation of the tibia approximated ± 15° like the native knee [30].

### Method for measuring the orientation of the tibia relative to the femoral component and recording anterior lift-off of the insert

After inserting trial components, the surgeon used the back of the wrist to lift the heel and passively extend the knee without applying an I–E moment to the tibia. The goniometric insert measured the angular tibial orientation relative to the line on the femoral component in degrees at extension (+ external/−internal). The surgeon reduced the patella, and the sub-vastus exposure stabilized the patella in the trochlear groove during flexion of the TKA to 90°. The heel was seated on a bump secured to the operating table, supporting the leg's weight. Without applying an I–E moment to the tibia, the surgeon recorded the tibial orientation of the insert goniometer relative to the femoral trial component and the occurrence of anterior lift-off as described by Scott [32]. When the insert trial lifted off the tibial baseplate anteriorly with knee flexion between 90° to 100°, the test was positive, and the PCL and flexion space were too tight [32]. Lift-off was not measured in millimeters as no published reports describe this refinement of Scott's method [2].

**Fig. 5:**
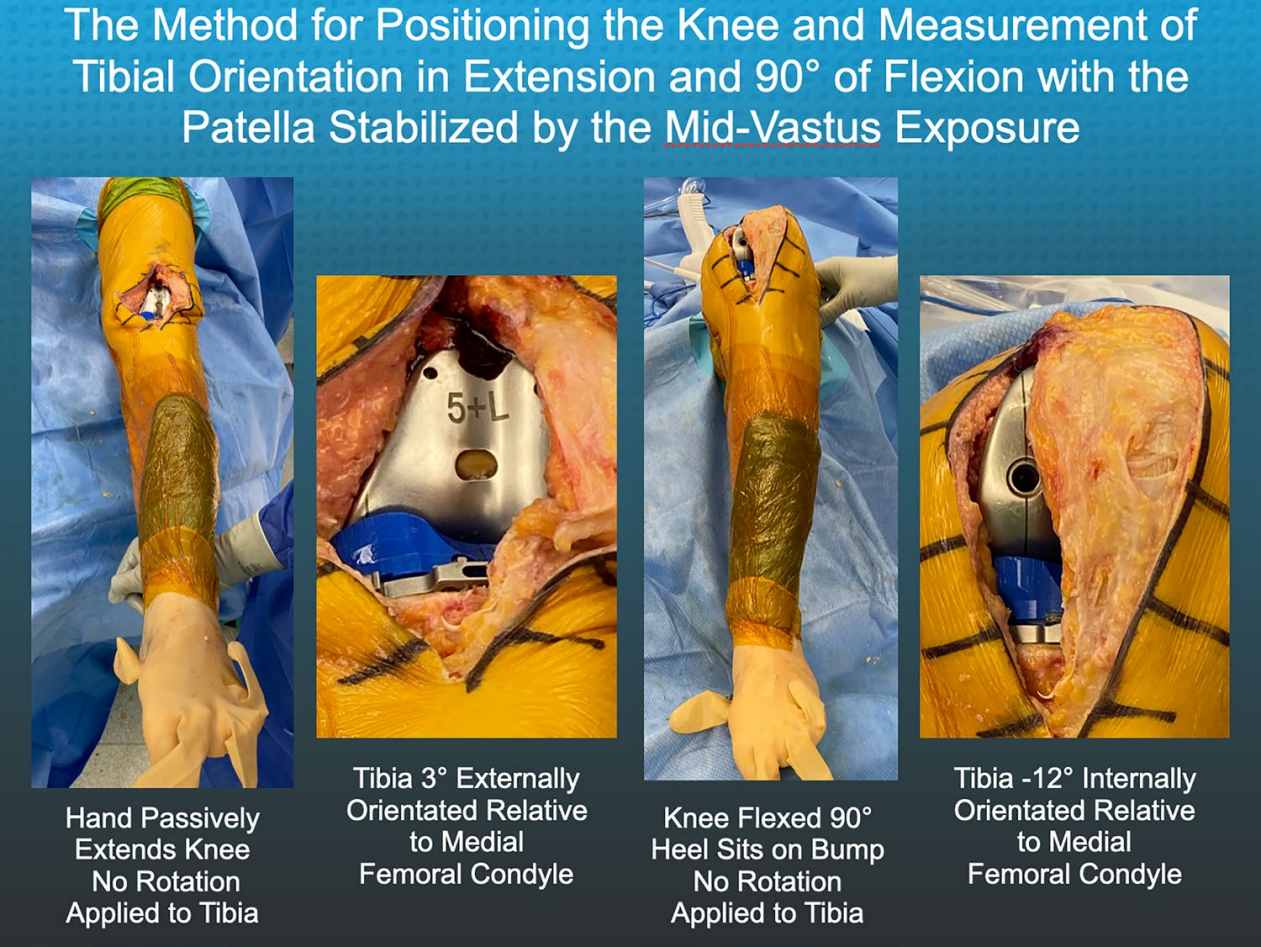
Composite shows the method for positioning the TKA and measuring the angular tibial orientation of the goniometer insert relative to the femoral component with the knee passively extended and with the TKA in 90° flexion with the heel resting on the bump supporting the leg without the surgeon applying rotation to the tibia

The heel was seated on a bump secured to the operating table without applying an I–E moment to the tibia. With the foot resting on the operating table and supporting the leg's weight, the surgeon recorded the tibial orientation at 90° flexion and the occurrence of anterior lift-off of the insert (Fig. 5). The surgeon recorded anterior lift-off as a dichotomous variable as either “absent” or “present”.

### Statistical analysis

Data were analyzed using statistical software (JMP^®^ Pro 15.2.1, www.jmp.com, SAS, Cary, NC, USA). The mean and standard deviation described the continuous variables. A Student’s paired *t* test determined whether internal rotation of the TKA was different between PCL retention and excision. The Chi-square test determined whether the incidence of anterior lift-off of the insert was different between PCL retention and excision. Significance was *p* < 0.05.

A senior academic bio-statistician recommended a repeatability and reproducibility analysis to characterize the measurement of tibial orientation relative to the femoral trial component with the insert goniometer. To characterize repeatability, a single surgeon measured the tibial orientation five times in four TKAs. The surgeon ranged the knee before measuring the tibial orientation in extension and 90° flexion. Repeatability in extension and at 90° flexion was computed as the pooled standard deviation and was 1°. To characterize reproducibility, two observers (SMH and AJN) measured the I–E orientation of the tibia at extension and 90° flexion in seven knees. A two-way random-effects model analysis of variance (ANOVA) computed the intraclass correlation coefficient (ICC). An ICC value of > 0.9 indicates excellent agreement, and 0.75–0.90 indicates good agreement. The ICC values of 0.89 for the measurement of tibial orientation at extension and 0.87 at 90° of flexion indicated good reproducibility.

## Results

**Fig. 6:**
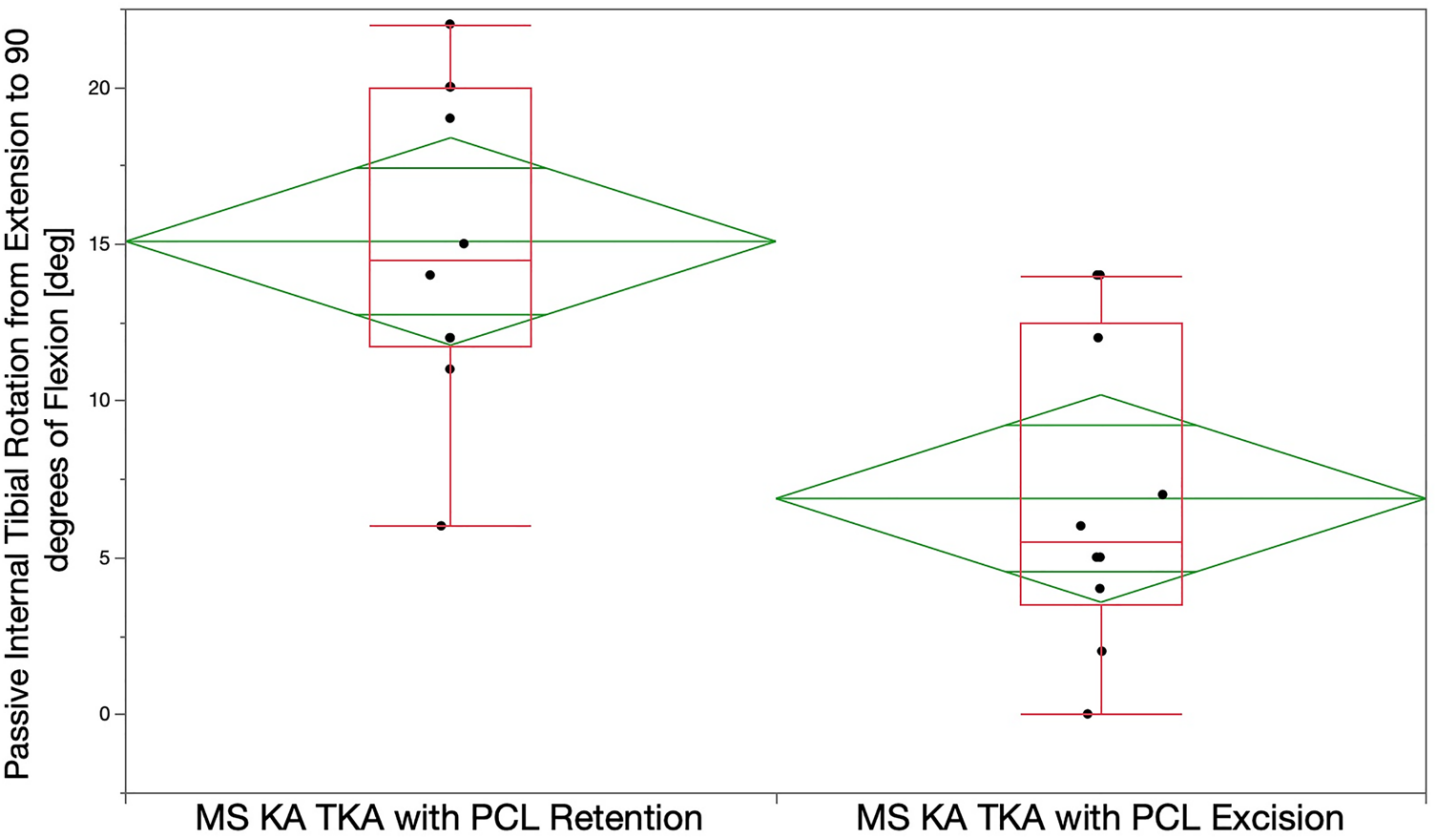
Box plots of ten cadaveric knees show the mean internal tibial rotation from maximum extension to 90° of flexion (transverse line in the middle of the green diamond) of 15° with PCL retention was significantly greater than the 7° after PCL excision after implantation of a medial ball-in-socket and lateral flat tibial insert TKA with unrestricted calipered KA (*p* < 0.0007). The top and bottom edges of the green diamond indicate the 95% confidence interval limits

Internal tibial rotation with PCL retention was greater than that with PCL excision. From extension to 90° flexion, the mean internal tibial rotation was 15 ± 5° vs. 7 ± 5° for PCL retention vs PCL excision (*p* < 0.0007) (Fig. 6). At 90° flexion, no TKA with PCL retention and one TKA with PCL excision had anterior lift-off of the insert (N.S.).

## Discussion

The most important findings from the present study were that retaining the PCL with an MP TKA implanted with unrestricted caliper verified KA restored higher internal tibial rotation between extension and 90° flexion and a negligible incidence of anterior lift-off of the insert when compared to PCL excision.

Although PCL retention is used sparingly with most MP designs because of concerns of limiting knee flexion, the present study showed that the PCL is needed to restore native internal tibial rotation and that its retention did not over-tension the flexion space. Similar to TKA, the function of the PCL in the native knee is crucial as cutting the PCL eliminates I–E tibial rotation [7]. Hence, promoting native knee internal tibial rotation also requires proper tension in the PCL. Fortunately, the restoration of native PCL tension is a direct consequence of unrestricted caliper verified KA TKA, as in vivo and in vitro studies reported restoration of native knee posterior laxity and medial and lateral tibial compartment forces [28, 29, 33–35].

Restoring native internal rotation about a medial pivot like the native knee requires the medial condyle of the femoral component to remain centered in the medial insert throughout the arc of motion, which may translate into favorable clinical results. A level 1 randomized-controlled trial reported that patients who underwent the medial constrained TKA scored significantly better on the Forgotten Joint Score and the quality-of-life subscale of the KOOS and KOOS-12 than those who underwent a CR-TKA [6]. These findings suggest that the medial constrained pivot knee is more likely to allow patients to "forget" the knee and restore their quality of life [6]. In vivo studies confirmed the medial pivot of the tibia during a deep knee bend and gait [1, 9, 31]. In addition, the lateral insert is flat to resemble the lack of lateral concavity in the native knee, which results in higher internal rotation than sagittal conforming lateral inserts with a posterior rim [1]. Thus, in the TKA, the posterior insert rim functions like a chock-block limiting internal tibial rotation [1]. In the native knee, the lateral meniscus does not limit internal tibial rotation since it falls off the posterior tibia in deep flexion [5, 17]. Hence, medial and lateral compartment articular geometry and PCL condition are critical factors governing tibial rotation relative to the femur [31].

While PCL resection has the detrimental effect of predictably increasing laxity in the flexion space an unpredictable amount in TKA, the present study identified another undesirable kinematic consequence: the loss of half of the internal tibial rotation relative to the native knee at 90° flexion. Like PCL resection in the native knee, no surgical strategy other than PCL reconstruction is likely to overcome the loss of internal tibial rotation from PCL resection in TKA and the concomitant risk of adverse clinical consequences such as patellar tilt, lateral displacement, and anterior knee pain [3, 7, 19].

The present cadaveric study has several limitations. The accuracy of the insert goniometer is specific for medial ball-in socket MP design and is not likely transferrable to a shallower and non-spherical MP design that allows medial A–P femorotibial displacement. The degree of internal tibial rotation is likely less for PS, PCL retaining, and ultra-congruent geometries, which have shallow medial and lateral insert concavities and a posterolateral rim that functions as a chock-block. The shallow medial concavity enables the femur to translate anteriorly and posteriorly, thereby lowering the PCL's tension necessary to drive internal tibial rotation. The degree of internal tibial rotation with PCL retention in a cadaveric knee in the present study could differ from in vivo values after unrestricted caliper verified KA TKA. Internal rotation values could change under different mechanisms of loading the TKA, such as weight-bearing activities.

Several factors could have affected the passive internal tibial rotation with PCL retention and excision. These factors included variance in the surgeon setting the slope of the tibial component which could tighten or loosen the flexion space and component size. However, because the study reported the difference in rotation within each knee specimen, these factors did not affect the primary conclusion that PCL retention restored more passive internal rotation than PCL excision.

## Conclusion

This preliminary study of ten cadaveric knees showed that PCL retention restored more passive internal tibial rotation than PCL excision with a negligible risk of anterior lift-off. However, in vivo analysis from multiple authors with a larger sample size is required to recommend PCL retention with an MP TKA design implanted with unrestricted caliper verified KA.
